# USING MULTIDIMENSIONAL SCALING IN COMBINATION WITH CLUSTER ANALYSIS TO STRUCTURE THE PELI-D QUESTIONNAIRE

**DOI:** 10.1093/geroni/igac059.702

**Published:** 2022-12-20

**Authors:** Johannes-Michael Bergmann, Tobias Stacke, Daniel Purwins, Christina Manietta, Mike Rommerskirch-Manietta, Armin Stroebel, Martina Roes

**Affiliations:** German Center for Neurodegenerative Diseases (DZNE), Göttingen, Nordrhein-Westfalen, Germany; Community Bergkamen, Bergkamen, Nordrhein-Westfalen, Germany; DZNE, Witten, Nordrhein-Westfalen, Germany; DZNE, Witten, Nordrhein-Westfalen, Germany; German Center For Neurodegenerative Diseases (DZNE), Witten, Nordrhein-Westfalen, Germany; Center for Clinical Studies, Erlangen, Bayern, Germany; German Center for Neurodegenerative Diseases (DZNE), Witten, Nordrhein-Westfalen, Germany

## Abstract

**Background:**

One way to contribute person-centered care is the use of a preference-based approach. In our project PELI-D the original PELI USA-version for Nursing Homes was (1) translated into German (2) and structured to explore construct validity of the instrument. Objective and Method: The PELI-D instrument (72-items) is used to illustrate how structuring procedures can be used for concept analysis. The approach allows to analyze the instrument from the perspective of the professional caregivers and to identify theoretical relations in further steps, which can be finally validated. For this purpose, the data of 58 professional nurses are analyzed.

**Results:**

We computed a three-dimensional concept map of preferences enabled us in a second step to identify an appropriate cluster solution. The five-cluster solution, which explained 79 percent of the total variance, seemed to offer the best balance between statistical analysis and detailed categorisation through qualitative interpretation.

